# Competency of improved artificial ecosystem optimizer in parameters identification of small and medium sized distribution transformers

**DOI:** 10.1038/s41598-025-14233-3

**Published:** 2025-09-12

**Authors:** Abdelmonem Draz, Hossam Ashraf, Ahmed R. El Shamy, Attia A. El-Fergany

**Affiliations:** 1 Electrical Power and Machines Department, Faculty of Engineering, Zagazig University, Zagazig, 44519 Egypt; 2 Department of Electrical Engineering and Information Systems, The University of Tokyo, Tokyo, 113-8656 Japan; 3 Advanced Power and Energy Center, Department of Electrical Engineering, Khalifa University, P. O. Box 127788, Abu Dhabi, UAE; 4 Faculty of Engineering, Zagazig University, Zagazig, 44519 Egypt

**Keywords:** Artificial ecosystem optimizer, Distribution transformers, Metaheuristic algorithms parameters estimation, Statistical analysis, Engineering, Electrical and electronic engineering

## Abstract

Accurate modelling of distribution transformers (TXs) is crucial to identify their operating characteristics across several power system applications. Consequently, this paper employs an improved version of the artificial ecosystem optimizer (called IAEO) in parameters estimation of distribution TXs with different four sizes (i.e. 4, 15, 112.5 and 167 kVA ratings). Comparison with well-known literature optimizers, including genetic algorithm, particle swarm optimizer, coyote optimization algorithm, artificial hummingbird optimizer, and others, validates the performance of the proposed IAEO. The IAEO demonstrates its superiority by getting the lowest possible value of the sum of absolute errors (SAEs) between measured and calculated values, which serves as the objective function (OF) to be optimized. Moreover, three additional optimizers are employed and compared to IAEO for all study cases: firefly algorithm, political optimizer, and exponential distribution optimizer. It is found that IAEO attains the minimum SAEs values of 1.12e-5 and 0.0322, outperforming the best competitors for 4 kVA and 15 kVA TXs, respectively. Furthermore, IAEO accurately captures the steady state fingerprint of all studied TXs in terms of efficiency and voltage regulation (VR). This way, the peak efficiency occurs at 36.2% loading in 112.5 kVA TX while the negative VR may reach -8% when the 167 kVA TX is loaded with its rated leading power factor. Finally, all executed optimizers are analyzed using several statistical indices, including t-test, where the proposed IAEO gets the smoothest and fastest OF minimization trend.

## Introduction

Transformers (TXs) are fundamental components in electrical power systems for securing efficient transmission and distribution of electricity through adjusting voltage levels as needed^1^. Therefore, ensuring their operational reliability and performance optimization is essential for maintaining power system stability^2^. Consequently, the development of accurate steady-state and dynamic models has become a major focus of recent research since such models can facilitate comprehensive analysis of TXs behavior across various operating conditions^3,4^. Specifically, through these models, key performance metrics, including efficiency, losses, and thermal response, can be accurately predicted prior to real-world implementation^5^. By reducing reliance on physical testing, these models help minimize development costs and enhance the design process. For example, understanding steady-state characteristics (CCs) such as impedance and voltage regulation aids system operators in making well-informed decisions related to maintenance, fault detection, and network planning. Additionally, as the integration of renewable energy sources continues to grow, the need for accurate TX models becomes even more pressing to ensure the infrastructure can adapt to evolving load demands and generation variability^6,7^.

Many TX’s models depend on parameters that are not explicitly provided in manufacturer datasheets^8^. The accuracy of these parameters directly affects the precision of simulation outputs in replicating real-world TX’s behavior. Thus, precise parameter identification is crucial to ensure such models accurately reflect TX’s performance under varying operating conditions^9^. Conversely, inadequate estimation methods can lead to inefficient design, increased operational expenses, and potential risks to system reliability. Therefore, selecting an appropriate determination technique is imperative for constructing robust models that facilitate effective monitoring, optimization, planning, and control of TX’s operations^10^.

Several challenges affect the accuracy of parameter determination, including harmonic distortion, transient effects, and core saturation^11^. Thus, various techniques have been employed, such as time-domain behavioral analysis^12^ and frequency response assessments^13^, to account for the aforementioned issues. Inrush current measurements have also been integrated into models to consider core saturation effects^14^. For parameter estimation initialization, phase measurement units, short-circuit and no-load tests, inrush current analysis, and load-based data acquisition are the commonly used measurement techniques^15^. However, performing these tests often requires taking TXs offline, which is impractical for large-scale implementation since it can lead to widespread power outages and increased economic losses^16^.

To overcome the aforementioned limitations, finite-element-based analytical techniques have been widely adopted for TX sizing^17,18^. Despite their effectiveness, these methods face difficulties when dealing with nonlinear materials and intricate TX structures. As a result, researchers have increasingly turned to metaheuristic optimization algorithms (MOAs). MOAs have demonstrated strong potential in solving a variety of engineering problems, such as relay coordination for TX protection^19^, parameter estimation in fuel cells^20^, photovoltaic (PV) systems^21^, and battery modelling^22^. MOAs have also been employed for economic dispatch^23^ and optimal placement of distributed energy resources^24^. Unlike traditional methods, MOAs can efficiently explore search spaces^25^, handle nonlinearities, and avoid local optima^26^, qualifying them as a well-suited technique for accurate and flexible parameter estimation even under challenging noise and uncertainties^27^. Their adaptability to different TX operating conditions further enhances their suitability for modern power systems, ultimately leading to improved energy management and distribution through precise TX modelling^28^.

In this regard, numerous studies have explored the application of MOAs for optimizing TX equivalent circuit parameter estimation by maximizing the correlation between computed and measured data^29^. This correlation is typically evaluated using a specific objective function (OF), where minimizing the OF value indicates superior algorithmic performance. Instead of relying on direct TX testing, researchers often extract real-world data from nameplate specifications or load-based measurements to enhance estimation accuracy without requiring service interruptions^30^. Various MOAs have been explored for transformer equivalent circuit parameter estimation, such as artificial bee colony (ABC) optimization, artificial hummingbird optimizer (AHO), blackhole optimization algorithm (BOA), coyote optimization algorithm (COA), chaotic optimization technique (COT), and crow search optimizer (CSO). Other MOAs applied in this domain include dandelion optimization algorithm (DOA), forensic-based investigation optimizer (FIO), generalized normal distribution optimizer (GNDO), genetic algorithm (GA), gravitational search algorithm (GSA), and hurricane optimization algorithm (HOA). Additionally, imperialistic competitive optimizer (ICA), improved Tasmanian devil optimizer (ITDO), jellyfish search optimizer (JSO), particle swarm optimizer (PSO), and slime mould optimizer (SMO) are attempted to attain the same goal.

Comprehensive comparisons of these MOAs are provided in Table 1, detailing their inspiration, OFs, advantages, disadvantages, TX types, and the specific tests used for circuit data extraction. The table categorizes different OFs applied to assess algorithmic accuracy, including percentage error (PE), sum of absolute error (SAE), average mean quadratic error (AMQE), mean quadratic error (MQE), sum of quadratic absolute percentage error (SQAPE), sum of quadratic error (SQE), and sum of quadratic relative error (SQRE). It also highlights the variations in TX types, from single-phase to three-phase transformers, and the corresponding tests such as no-load, short-circuit, full-load, load, DC, and nameplate data extraction. Despite their advantages, MOAs require careful parameter tuning, as improper adjustments may lead to suboptimal results. Their inherently stochastic nature can sometimes result in inconsistent solutions, necessitating the application of multiple optimization strategies to enhance robustness. Furthermore, as per the no free lunch theorem, no single optimization algorithm is universally superior for all parameter estimation tasks, reinforcing the need for diverse approaches tailored to specific problem complexities^15,16,31,32^.

**Table 1:** Comparative analysis of MOAs for transformer parameters estimation.

MOA	Inspiration	OF	Advantages	Disadvantages	TX type	Tests for circuit data
ABC^29^	Swarm-based algorithm mimicking foraging behaviour of honeybees	PE	Good global search capability, easy to implement, robust against local optima	Slow convergence, may require fine-tuning for exploitation	1-ph	✓ No-load test ✓ Short-circuit test
AHO^30^	Inspired by hummingbird foraging and migration behaviour	SAE	Effective in handling complex and dynamic problems, strong adaptability	Requires fine-tuning for optimal performance	1-ph	✓ Full-load test
BOA^3^	Population-based approach inspired by blackhole dynamics	AMQE	Fast convergence rate, effective for multimodal problems	Risk of premature convergence, sensitive to initial conditions	1-ph	✓ Full-load test
COA^28^	Based on the social behaviour of coyotes	SQAPE	Balances exploration and exploitation, adaptable to various problems	Performance depends on parameter selection	1-ph 3-ph	✓ No-load test ✓ Short-circuit test ✓ DC test
COT^34^	Uses chaotic maps to guide search process	SQE	Enhances global search, avoids local optima	High sensitivity to chaotic map selection	1-ph	✓ Nameplate data ✓ Load test ✓ No-load test ✓ Short-circuit test ✓ Full-load test
CSO^35^	Mimics intelligence and memory of crows	MQE	Strong exploratory ability, memory-based learning	Convergence speed may be slow	1-ph	✓ Nameplate data
DOA^15^	Inspired by the dispersal behaviour of dandelion seeds	SQRE	Good for multimodal problems, maintains diversity	Requires parameter tuning for efficiency	1-ph 3-ph	✓ Load test
FIO^36^	Inspired by forensic crime-solving technique	PE	Strong exploitation ability, effective in parameter tuning	May struggle with high-dimensional search spaces	1-ph	✓ Full-load test
GND^37^O	Based on statistical normal distribution properties	MQE	Robust performance in optimization problems	Computationally expensive	1-ph	✓ Load test
GOA^38^	Evolutionary algorithm based on natural selection	SQE	Well-studied, widely used, effective for global search	Slow convergence rate, risk of premature convergence	1-ph	✓ Nameplate data
GSA^39^	Inspired by Newton’s laws of gravity	SQE	Effective in handling high-dimensional problems	Computationally expensive, slow convergence	1-ph	✓ Nameplate data
HOA^40^	Mimics hurricane eye formation and behaviour	MQE	Effective global search strategy, good exploration	May require additional constraints for fine-tuning	1-ph	✓ No-load test ✓ Short-circuit test
ICO^39^	Based on imperialistic competition dynamics	SQE	Fast convergence, strong global search	Risk of getting trapped in local optima	1-ph	✓ Nameplate data
ITDO^16^	Inspired by Tasmanian devil hunting behaviour	SQRE	Efficient in multimodal search, robust exploration	Computational complexity increases with problem size	1-ph	✓ No-load test ✓ Short-circuit test ✓ DC test ✓ Load test
JSO^41^	Mimics jellyfish movement patterns	NR	Well-balanced exploration and exploitation	Requires careful parameter setting	1-ph	✓ Nameplate data
PSO^38^	Swarm-based approach inspired by bird flocking	SQE	Fast convergence, easy implementation	Prone to premature convergence, sensitive to parameter tuning	1-ph	✓ Nameplate data
SMO^42^	Based on slime mould’s oscillatory foraging behaviour	SQRE	Adaptive, good balance between exploration and exploitation	May struggle with parameter selection	1-ph 3-ph	✓ Load test

“NR” means “not reported” in the referred reference.

Modifications of MOAs are often introduced to enhance their performance in terms of convergence speed, solution quality, or robustness across diverse optimization problems^43^. These enhancements are necessary because the original or classical versions may suffer from issues like premature convergence, slow exploration, or stagnation in local optima^44^. Modifications are typically applied to core operators such as initialization, selection, crossover, mutation, and update mechanisms. For instance, hybridizing exploration and exploitation strategies, incorporating adaptive parameters, or using problem-specific knowledge can significantly improve algorithmic efficiency. Overall, such modifications aim to strike a better balance between global search and local refinement, thereby increasing the algorithm’s generalization capability^45^.

Driven by the ongoing need for accurate transformer models in system design and operation, the limitations of direct testing methods, and the growing demand for accurate, non-intrusive parameter estimation methods compatible with modern grid requirements, this study explores the improved artificial ecosystem optimizer (IAEO), a recent variant of the artificial ecosystem optimizer (AEO), for TXs parameter estimation^46^. Compared to earlier MOAs, IAEO offers better convergence stability, enhanced global search capabilities, and fewer parameter dependencies, making it a strong candidate for complex nonlinear optimization tasks such as TX parameter estimation. The IAEO has demonstrated promising results in other power system applications, indicating its potential for handling complex transformer modeling problems^47,48^. This study aims to investigate the application of IAEO for offline transformer parameter estimation, evaluating its effectiveness across various transformer sizes and a wide range of operating conditions. To validate its performance, the proposed method is benchmarked against several recent metaheuristic optimization algorithms from the literature. Accordingly, the contributions of this research can be summed up as follows: (i) Utilizing an improved IAEO in parameters determination of distribution TXs, (ii) Validating IAEO competency against literature algorithms against well-established algorithms from the literature, including firefly algorithm (FA), political optimizer (PO), and exponential distribution optimizer (EDO), (iii) Evaluating the proposed methodology over small and medium sized single-phase TXs, (iv) Identifying the rated fingerprint of these TXs under various loading conditions, and (v) Assessing the proposed methodology with several statistical performance measures.

The remainder of this research is structured as follows: Mathematical modelling of single-phase TXs with governing equations are presented in Section “Transformer mathematical modelling”. The general procedure of the proposed IAEO with its methodology are discussed in Section “Optimization technique”. Section “Optimization model formulation” formulates the optimization problem with associated constraints and details the statistical analyses methods employed for testing different MOAs algorithms. Section “Simulation results with debates” provides simulation results, illustrative graphs, and discussions. Finally, Section “Conclusions” concludes the research work along future insights.

## Transformer mathematical modelling

**Fig. 1:**
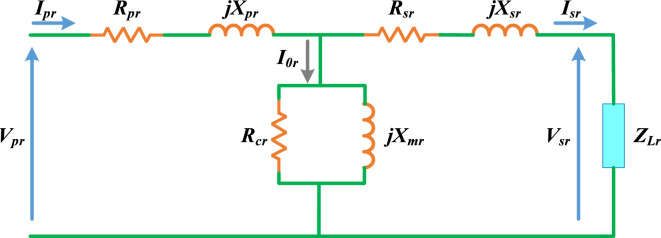
Transformer equivalent circuit per phase.

TXs are composed of primary and secondary windings that are magnetically linked through an iron core. The primary winding is connected to the supply voltage, ${V}_{pr} \left(V\right)$, while the secondary winding, ${V}_{sr} \left(V\right)$, delivers power to the load, ${Z}_{Lr} \left(\Omega \right)$. Consequently, the TX equivalent circuit incorporates primary and secondary resistances, ${R}_{pr}$ and ${R}_{sr}$, primary and secondary leakage reactances, ${X}_{pr}$ and ${X}_{sr}$, core-loss resistance, ${R}_{cr}$, and magnetizing reactance, ${X}_{mr}$, all measured in $\left(\Omega \right)$. Notably, all these parameters are referenced to the primary side, as illustrated in Fig. 1^15,33^.

Based on this configuration, the impedances associated with the primary and secondary windings, as well as the core magnetizing branch, denoted as ${Z}_{pr}$, ${Z}_{sr}$, and ${Z}_{mr}$, respectively, all in *Ω*, can be mathematically expressed as follows:

1 $${Z}_{pr}={R}_{pr}+j{X}_{pr}$$

2 $${\text{Z}}_{\text{sr}}={\text{R}}_{sr}+{\text{jX}}_{sr}$$

3 $${Z}_{mr}=\frac{{R}_{cr}\times j{X}_{mr}}{{R}_{cr}+j{X}_{mr}}$$

When the TX is energized by an applied voltage source ${V}_{pr}$ and connected to a load ${Z}_{Lr}$, the overall impedance observed from the primary side, ${Z}_{all} \left(\Omega \right)$, the input current, ${I}_{pr} \left(A\right)$, the load current, ${I}_{sr} \left(A\right)$, and the load voltage, ${V}_{sr}$, can be derived using (4) to (7)^29^:

4 $${Z}_{all}={Z}_{pr}+\frac{{Z}_{mr}\times \left({Z}_{Lr}+{Z}_{sr}\right)}{{Z}_{mr}+{Z}_{Lr}+{Z}_{sr}}$$

5 $${I}_{pr}=\frac{{V}_{pr}}{{Z}_{all}}$$

6 $${I}_{sr}=\frac{{Z}_{mr}}{{Z}_{mr}+{Z}_{Lr}+{Z}_{sr}}\left(\frac{{V}_{pr}}{{Z}_{allr}}\right)$$

7 $${V}_{sr}={Z}_{sr}\times {I}_{sr}$$

Under typical operating conditions, the TX percentage voltage regulation, $VR\%$, input power, ${P}_{inr} (W)$, output power, ${P}_{outr} (W)$, and efficiency, $\eta (\%)$, can be determined as indicated in (8)-(11)^34,40^:

8 $$VR\%=\frac{\left|{V}_{pr}\right|-\left|{V}_{sr}\right|}{\left|{V}_{pr}\right|}\times 100$$

9 $${P}_{inr}=real\left\{{V}_{pr}\times {I}_{pr}^{*}\right\}$$

10 $${P}_{outr}=real\left\{{V}_{sp}\times {I}_{sp}^{*}\right\}$$

11 $$\eta \%=\frac{{P}_{outr}}{{P}_{inr}}\times 100$$

Accordingly, it is clear that ${R}_{pr}$, ${X}_{pr}$, ${R}_{cr}$, ${X}_{mr}$, ${R}_{sr}$, and ${X}_{sr}$ need to be optimally estimated to ensure an accurate TX model that aligns with actual measured data. Once these parameters are identified, the TX equivalent impedance in per unit (PU), denoted as ${Z}_{TXr}$, can be computed by (12), which will be further utilized in short-circuit (SC) calculations. Lastly, the loading level at which the TX achieves maximum efficiency (ε) can be determined by (13).

12 $${Z}_{TXr}={Z}_{all}\times \frac{{{V}_{ps}}^{2}}{{S}_{TXr}}$$

13 $$\varepsilon =\sqrt{\frac{{P}_{core}}{{P}_{cufl}}} \times 100$$

where, ${S}_{TXr}$ represents the TX apparent power in $VA$, ${P}_{core}$ accounts for iron or core losses which remain constant regardless of loading percentage in $W$, and ${P}_{cufl}$ signifies the copper losses in $W$ when the TX is fully loaded.

## Optimization technique

This section introduces the inspiration and equations of the IAEO, explains the differences and advantages compared to the conventional AEO, and describes how it performs the exploration, and exploitation phases and makes the transition between them.

### Conceptual foundation

The AEO is a nature-inspired algorithm based on the dynamics of ecosystems, where individuals represent solutions, interact with each other and their environment. The algorithm takes inspiration from the natural process of evolution, where species must adapt to their surroundings, optimize their survival strategies, and maximize their chances of reproducing. This process is modeled in the AEO, where the algorithm’s population represents species in an ecosystem, and various operations like production, consumption, and decomposition mimic natural behaviors that drive the search for optimal solutions^48^. The AEO employes three main evolutionary operators: production, consumption, and decomposition, which simulate the survival and reproduction strategies of species in a natural ecosystem. These operators work together to guide the algorithm through the exploration and exploitation phases of the optimization process^47^.

#### Production phase

The production operator in AEO is inspired by the way organisms reproduce and generate offspring. In this operator, the best individual in the population produces new solutions by combining its traits with those of a randomly selected individual. This process aims to introduce new individuals into the population, contributing to the search for better solutions. The new candidate solution produced at iteration $it+1$, ${X}_{1}\left(it+1\right)$, is generated as:

14 $${X}_{1}\left(it+1\right)=\left(1-\alpha \right)\times {X}_{{N}_{p}}\left(it\right)+\alpha \times {X}_{rd}\left(it\right)$$

where, ${X}_{{N}_{p}}\left(it\right)$ represents the current best solution in the population ${N}_{p}$ at iteration $it$. $\alpha$ is a production factor that is gradually decreased during the optimization process to reduce exploration and enhance exploitation, as defined in (15). ${X}_{rd}$ is a randomly selected individual from the population at iteration $it$ which is calculated by (16).

15 $$\alpha =\left(1-\frac{it}{{IT}_{m}}\right)\times {s}_{1}$$

16 $${X}_{rd}=s\times \left({U}_{l}-{L}_{l}\right)+{L}_{l}$$

where, ${IT}_{m}$ is the maximum number of iterations. ${U}_{l}$ and ${L}_{l}$ denote the higher and lower limits of the search space, respectively. ${s}_{1}$ and s are randomly generated number and vector, respectively. ${s}_{1}$ and s values range from $0$ to $1$.

The production operator helps to introduce diversity in the population by generating new individuals. The value of $\alpha$ decreases over time, shifting the focus from global search (exploration) to local search (exploitation).

#### Consumption phase

The consumption operator models the interaction between individuals in the ecosystem based on their fitness levels. Individuals can consume other individuals based on their relative fitness, mimicking the behavior of herbivores, carnivores, and omnivores in nature. This operator helps in refining the search by allowing individuals to consume solutions that are better or more competitive. General consumption behavior is controlled by the consumption factor, $C$, based on Levy flight, as defined by (17) and (18).

17 $$C=0.5\times \frac{{v}_{1}}{\left|{v}_{2}\right|}$$

18 $${v}_{1}\sim \widetilde{N}\left(\text{0,1}\right), { v}_{2}\sim \widetilde{N}\left(\text{0,1}\right)$$

where, $\widetilde{N}\left(\text{0,1}\right)$ denotes a Gaussian distribution with a zero mean and unit variance.

The consumption phase is modeled by three different consumers:

- An herbivore consumes the nearest producer, a solution with higher fitness, which is mathematically expressed by (19).
- A carnivore consumes other randomly selected individuals in the population based on their fitness, as given by (20).
- An omnivore consumes both producers and other consumers, enabling further diversity in the population, as explained in (21).
  19 $${X}_{i}\left(it+1\right)={X}_{i}\left(it\right)+C.\left({X}_{i}\left(it\right)-{X}_{1}\left(it\right)\right), i\in \left(2,\dots ,{N}_{p}\right)$$
  20 $${X}_{i}\left(it+1\right)={X}_{i}\left(it\right)+C.\left({X}_{i}\left(it\right)-{X}_{j}\left(it\right)\right), i\in \left(3,\dots ,{N}_{p}\right) \& j=rand\left(\left[2, i-1\right]\right)$$
  $${X}_{i}\left(it+1\right)={X}_{i}\left(it\right)+C.\left({s}_{2}\left({X}_{i}\left(it\right)-{X}_{j}\left(it\right)\right)+\left(1-{s}_{2}\right)\left({X}_{i}\left(it\right)-{X}_{j}\left(it\right)\right)\right),$$
  21 $$i\in \left(3,\dots ,{N}_{p}\right) \& j=rand\left(\left[2, i-1\right]\right)$$

where ${X}_{i}\left(it+1\right)$ is the current position of the ${i}^{th}$ individual. ${X}_{j}\left(it\right)$ is a randomly chosen individual from earlier individuals, excluding the best. ${s}_{2}$ is a randomly generated scalar from 0 to 1.

#### Decomposition phase

The decomposition operator simulates the death of individuals in the ecosystem, where deceased individuals are decomposed, and their CCs contribute to the survival of the remaining individuals. This operator ensures diversity within the population and avoids the algorithm from becoming stuck in local optima. The mathematical representation of the decomposition phase is given by (22).

22 $${X}_{i}\left(it+1\right)={X}_{n}\left(it\right)+D.\left({e.X}_{n}\left(it\right)-{h.X}_{i}\left(it\right)\right), i\in \left(1,\dots ,{N}_{p}\right)$$

where, ${X}_{n}\left(it\right)$ is the best individual at iteration $it$. $D$ and $e$ are random factors that represent the decomposition strength and determine the direction of the decomposition, which are described by (23), (24), respectively. $h$ a random factor that influences the strength of the decomposition, given by (25).

23 $$D=3\times \widetilde{N}\left(\text{0,1}\right)$$

24 $$e={s}_{3}\times.\:randi\left(\left[1\:\: 2\right]\right)-1$$

25 $$h=2{s}_{3}-1$$

where, ${s}_{3}$ is a randomly generated number between (0, 1).

This formulation enables the best individual to contribute its traits to the rest of the population in a directionally randomized manner, thereby enhancing exploration capabilities while retaining information from superior solutions.

### IAEO

**Fig. 2:**
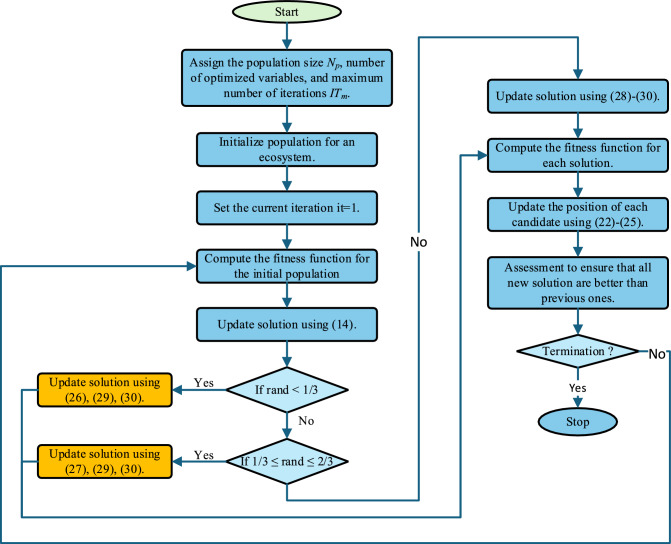
Flowchart of the IAEO.

The IAEO improves upon the traditional AEO by refining its performance. Unlike the AEO, which functions across three phases, production, consumption, and decomposition, the IAEO specifically targets and optimizes the efficiency of the consumption phase. The mathematical formulas of the consumption phase (19)-(21) are upgraded to a sinusoidal-based ones. This method generates diverse solutions by oscillating outward or towards the optimal solution. Accordingly, it contributes to a more dynamic and effective search process. Specifically, the mathematical representation of the herbivore, carnivore, and omnivore consumers are updated to that in (26,27,28,29,30), respectively^48^.

26 $${X}_{i}\left(it+1\right)=\left\{\begin{array}{c}{X}_{i}\left(it\right)+{s}_{4}.\text{sin}\left({s}_{5}\right).C.\left({X}_{i}\left(it\right)-{X}_{1}\left(it\right)\right), if {s}_{6}<0.5, i\in \left(2,\dots ,{N}_{p}\right)\\ {X}_{i}\left(it\right)+{s}_{4}.\text{cos}\left({s}_{5}\right).C.\left({X}_{i}\left(it\right)-{X}_{1}\left(it\right)\right), if {s}_{6}\ge 0.5, i\in \left(2,\dots ,{N}_{p}\right)\end{array}\right.$$

27 $${X}_{i}\left(it+1\right)=\left\{\begin{array}{c}{X}_{i}\left(it\right)+{s}_{4}.\text{sin}\left({s}_{5}\right).C.\left({X}_{i}\left(it\right)-{X}_{j}\left(it\right)\right), {s}_{6}<0.5, i\in \left(3,\dots ,{N}_{p}\right)\\ {X}_{i}\left(it\right)+{s}_{4}.\text{cos}\left({s}_{5}\right).C.\left({X}_{i}\left(it\right)-{X}_{j}\left(it\right)\right), {s}_{6}\ge 0.5, i\in \left(3,\dots ,{N}_{p}\right)\\ j=rand\left(\left[2, i-1\right]\right)\end{array}\right.$$

28 $${X}_{i}\left(it+1\right)=\left\{\begin{array}{c}{X}_{i}\left(it\right)+{s}_{4}.\text{sin}\left({s}_{5}\right).C.\left({s}_{2}\left({X}_{i}\left(it\right)-{X}_{j}\left(it\right)\right)+\left(1-{s}_{2}\right)\left({X}_{i}\left(it\right)-{X}_{j}\left(it\right)\right)\right), {s}_{6}<0.5, i\in \left(3,\dots ,{N}_{p}\right)\\ {X}_{i}\left(it\right)+{s}_{4}.\text{cos}\left({s}_{5}\right).C.\left({s}_{2}\left({X}_{i}\left(it\right)-{X}_{j}\left(it\right)\right)+\left(1-{s}_{2}\right)\left({X}_{i}\left(it\right)-{X}_{j}\left(it\right)\right)\right), {s}_{6}\ge 0.5, i\in \left(3,\dots ,{N}_{p}\right)\\ j=rand\left(\left[2i-1\right]\right)\end{array}\right.$$

29 $${s}_{4}=2-It\left(\frac{2}{{IT}_{m}}\right)$$

30 $${s}_{5}=2\pi \times rand(\text{0,1})$$

where ${s}_{4}$ controls the step size reduction over time and ${s}_{5}$ introduces a random phase angle. ${s}_{6}\in (\text{0,1})$ is random scalar to switch between sine and cosine operations. By improving the consumption phase through the sine–cosine method, it enhances the algorithm’s ability to generate diverse solutions and more effectively explores the search space. This approach helps maintain a balance between exploration and exploitation which prevents premature convergence. Additionally, the dynamic nature of the IAEO allows for better adaptation to complex optimization problems since it improves both convergence speed and solution quality. The flowchart of the IAEO is presented in Fig. 2.

Time complexity is a crucial metric for evaluating an algorithm’s performance, as it reflects both operational efficiency and the capacity to address complex optimization problems. The time complexity analysis for AEO and its improved variant, IAEO, is detailed below:

For AEO, given a population size N, problem dimension D, and maximum number of iterations T, the time complexities are as follows:

- Population initialization: O (N.D)
- Fitness evaluation: O (N.D)
- Producer position update: O (D)
- Consumer position update: O ((N-1).D)
- Decomposer position update: O (T.N.D)

Thus, the overall time complexity of AEO is dominated by O (T.N.D).

For IAEO, which uses the same parameters as AEO, the complexities are:

- Population initialization via Latin Hypercube Sampling (LHS): O (N.D)
- Quadratic interpolation: O (T.N.D)
- Adaptive neighborhood search: best-case complexity is O (0), while worst-case is O (T.N.D)

Hence, the overall time complexity of IAEO remains O (T.N.D). Although IAEO incorporates additional mechanisms, its time complexity does not increase exponentially compared to AEO. However, it yields significantly better performance.

## Optimization model formulation

### Objective function

The OF acts as the key performance indicator to quantify the mismatch between the actual measured data and the values produced by the model. Hence, diminishing its value signifies the optimizer ability to fine-tune TX parameters, resulting in a close alignment between the model outcomes and actual behavior. A well-defined OF not only directs the MOA toward precise solutions but also enhances its ability to handle complex and nonlinear CCs inherent in TX modelling. Thus, the efficiency of an MOA in parameter estimation largely depends on how accurately the OF captures the problem’s constraints and the nature of the data. Consequently, the SAE between the actual and computed datasets is selected as the objective criterion, as defined in (31). The mathematical representation of the proposed OF is provided in(32).

31 $$SAE=\left|{I}_{pr}-{I}_{prms}\right|+\left|{I}_{sr}-{I}_{srms}\right|+\left|{V}_{sr}-{V}_{srms}\right|+\left|\eta -{\eta }_{ms}\right|$$

32 $$OF=minimize \left(SAE\right)$$

where ${I}_{prms}$, ${I}_{srms}$, and ${V}_{srms}$ represent the actual measured values of the primary current, load current, and load voltage, respectively. ${\eta }_{ms}$ denotes the recorded TX efficiency corresponding to a given loading condition. Inequality constraints defining the boundaries of the optimized variables are formulated in (33).

33 $$\text{Subject to}\left\{\begin{array}{c}{{R}_{pr,min}\le R}_{pr}\le {R}_{pr,max}\\ {{R}_{sr,min}\le R}_{sr}\le {R}_{sr,max}\\ {{X}_{pr,min}\le X}_{pr}\le {X}_{pr,max}\\ {{X}_{sr,min}\le X}_{sr}\le {X}_{sr,max}\\ {{R}_{cr,min}\le R}_{cr}\le {R}_{cr,max} \\ {{X}_{mr,min}\le X}_{mr}\le {X}_{mr,max}\end{array}\right.$$

This formulation ensures that the optimized parameters remain within their predefined physical limits, improving the reliability and feasibility of the estimated TX model. Furthermore, several OFs may also be examined to ensure the robustness of the proposed methodology such as mean absolute error (MAE) and mean square error (MSE) which can be formulated as shown in (34) and (35) respectively.

34 $$MAE=\frac{1}{3}\left\{\left|{I}_{pr}-{I}_{prms}\right|+\left|{I}_{sr}-{I}_{srms}\right|+\left|{V}_{sr}-{V}_{srms}\right| \right\}$$

35 $$MSE=\frac{1}{2}\left\{{\left|\frac{{I}_{pr}-{I}_{prms}}{{I}_{prms}}\right|}^{2}+{\left|\frac{{I}_{sr}-{I}_{srms}}{{I}_{srms}}\right|}^{2}+{\left|\frac{{V}_{sr}-{V}_{srms}}{{V}_{srms}}\right|}^{2} \right\}$$

### Statistical analysis

A t-test, or Student’s t-test, is a statistical method used in hypothesis testing to compare the means of one or two populations. It can be applied in various scenarios: To assess if the mean of a single group differs from a specified value (one-sample t-test), to determine if there is a difference between the means of two independent groups (independent two-sample t-test), or to evaluate whether there is a significant difference between paired measurements (paired, or dependent samples t-test). First, the hypothesis to be tested is defined and an acceptable level of risk for making an incorrect conclusion is chosen. For instance, when comparing two populations, one may hypothesize that their means are equal and set a threshold for the likelihood of mistakenly concluding there’s a difference when there isn’t. Then, a test statistic ($ts$) based on the data is calculated and compared to a critical value from the t-distribution. Based on this comparison, one either rejects or fails to reject the null hypothesis.

When defining a hypothesis, one also should determine whether to use a one-tailed or two-tailed test. This decision should be made before gathering data or performing any calculations. It applies to all three types of t-tests for means. For all t-tests involving means, the analysis follows the same steps:

1. Start by defining null and alternative hypotheses before gathering any data.
2. Select an alpha (α) value, which represents the level of risk possible to accept for making an incorrect conclusion. For instance, if α is set to 0.05 when comparing two independent groups, it is accepted that a 5% chance of incorrectly concluding that the population means differ when they actually don’t.
3. Review the data for any errors.
4. Verify that the assumptions required for the test are met.
5. Conduct the test and draw a conclusion. All t-tests for means involve calculating a $ts$, which is then compared to a theoretical value from the t-distribution. This theoretical value depends on both the α value and the degrees of freedom, $df$, in the data.

A one-sample t-test is a statistical hypothesis test used to assess whether the mean of an unknown population differs from a specified value. Initially, the mean, standard deviation, SD, and sample size, n, are defined, then, the standard error of the mean $, {SE}_{mean}$, is computed from (36).

36 $${SE}_{mean}=\frac{\text{SD}}{\sqrt{n}}$$

$ts$ can be calculated through (37):

37 $$ts=\frac{\Delta }{{SE}_{mean}}$$

where $\Delta$ denotes the difference between the sample mean and the assumed value (hypothesis theory). To make the decision, the $ts$ value is compared to a value from the t-distribution. This process involves two steps:

1. The level of accepted risk is determined by mistakenly identifying a difference when none exists. As mentioned earlier, take a 5% chance of concluding that the unknown population mean differs from zero when it actually doesn’t. In statistical terms, we set α = 0.05. Ideally, this risk level (α) should be established before collecting any data.
2. The corresponding value from the t-distribution is obtained based on the risk level. For a t-test, this requires knowing the $df$, which are determined by the sample size as illustrated in (38).
  38 $$df=n-1$$

## Simulation results with debates

Initially, the competitiveness of the proposed IAEO against the original AEO is checked in solving the CEC2017 benchmark functions. Table 2 lists a brief comparison among selected test suits before going through the research’s core. Afterwards, the efficacy of the proposed IAEO is verified either with published work or newly executed algorithms over the nominated approach. Four different study cases are investigated in this paper: 4 kVA^30,49^, 15 kVA^30,49^, 112.5 kVA^50^, and 167 kVA^35^. For the first two cases, IAEO will be compared with the acclaimed GA^38,51^, PSO^36,51^, GSA^39^, and ICA^39^. In addition, JSO^41^ and FIO^36^ are referred in 4 kVA TX while COA^39^ and AHO^30^ are reviewed in the 15 kVA test case. Concurrently, 3 algorithms are executed with the main IAEO: FA^52^, PO^53^, and EDO^54^ in addition to the original AEO to manifest how the modifications improve the simulation results.

**Table 2:** AEO vs IAEO in solving the CEC2017 test suite.

Function	Item	IAEO	AEO
**F3**	Best	2.12e + 3	7.32e + 4
	Mean	5.81e + 3	8.90e + 4
	SD	2.10e + 3	1.16e + 4
	CPU time (s)	0.4760	0.2840
**F4**	Best	4.63e + 2	1.32e + 3
	Mean	5.11e + 2	2.23e + 3
	SD	2.60e + 1	7.45e + 2
	CPU time (s)	0.4960	0.3020

It is worth highlighting that the formulated OF announced in Section"Optimization model formulation"is utilized in all study cases with the extension of the efficiency supplement in the first case. Clearly, all the implemented algorithms are executed with the same conditions for an equitable comparison: 100 populations with 200 iterations for the 1^st^ two cases and 400 iterations for the other two cases with 10 independent simulation runs for all cases. Furthermore, lower boundaries (LB) and upper boundaries (UB) of the modelled TXs are listed in Table 3 as addressed in the literature. Last but not least, all results have been rounded to four decimal points for improved clarity and convenience.

**Table 3:** Lower and upper boundaries of the TXs decision variables.

**LB**	${R}_{pr,min}$	${R}_{sr,min}$	${X}_{pr,min}$	${X}_{sr,min}$	${R}_{cr,min}$	${X}_{mr,min}$
Case 1: 4 kVA	0.1	0.1	0.1	0.1	1000	100
Case 2: 15 kVA	0.1	0.1	0.1	0.1	100,000	1000
Case 3: 112.5 kVA	30	30	100	100	200,000	40,000
Case 4: 167 kVA	30	30	100	100	200,000	40,000

**UB**	${R}_{pr,max}$	${R}_{sr,max}$	${X}_{pr,max}$	${X}_{sr,max}$	${R}_{cr,max}$	${X}_{mr,max}$
Case 1: 4 kVA	3	4	3	4	2000	1000
Case 2: 15 kVA	5	5	5	5	200,000	10,000
Case 3: 112.5 kVA	70	70	300	300	400,000	100,000
Case 4: 167 kVA	70	70	300	300	400,000	100,000

### Study case 1: 4 kVA

**Fig. 3:**
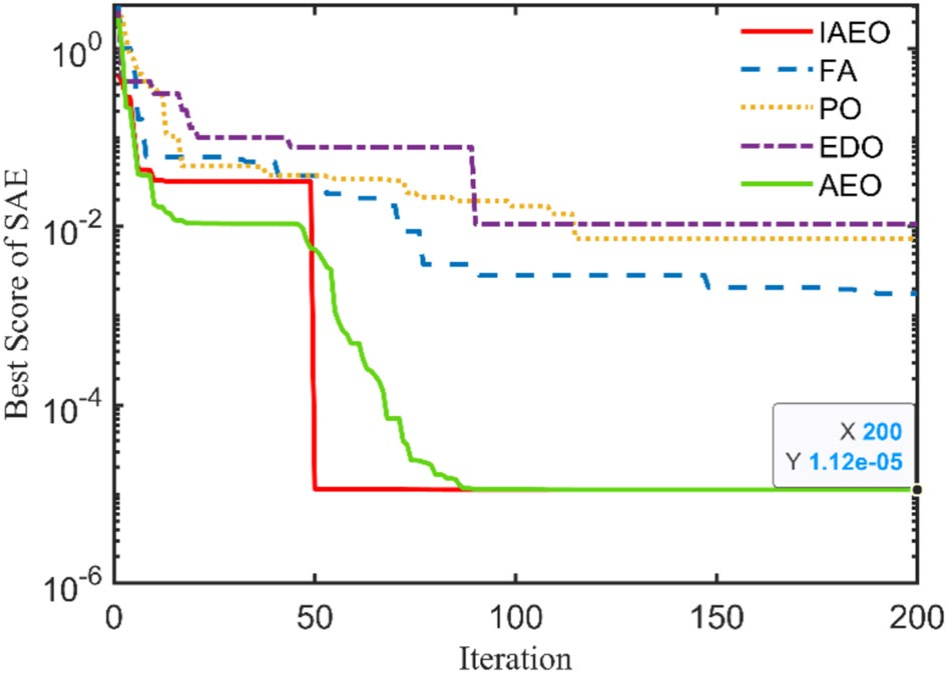
OF convergence trend of 4 kVA TX.

**Fig. 4:**
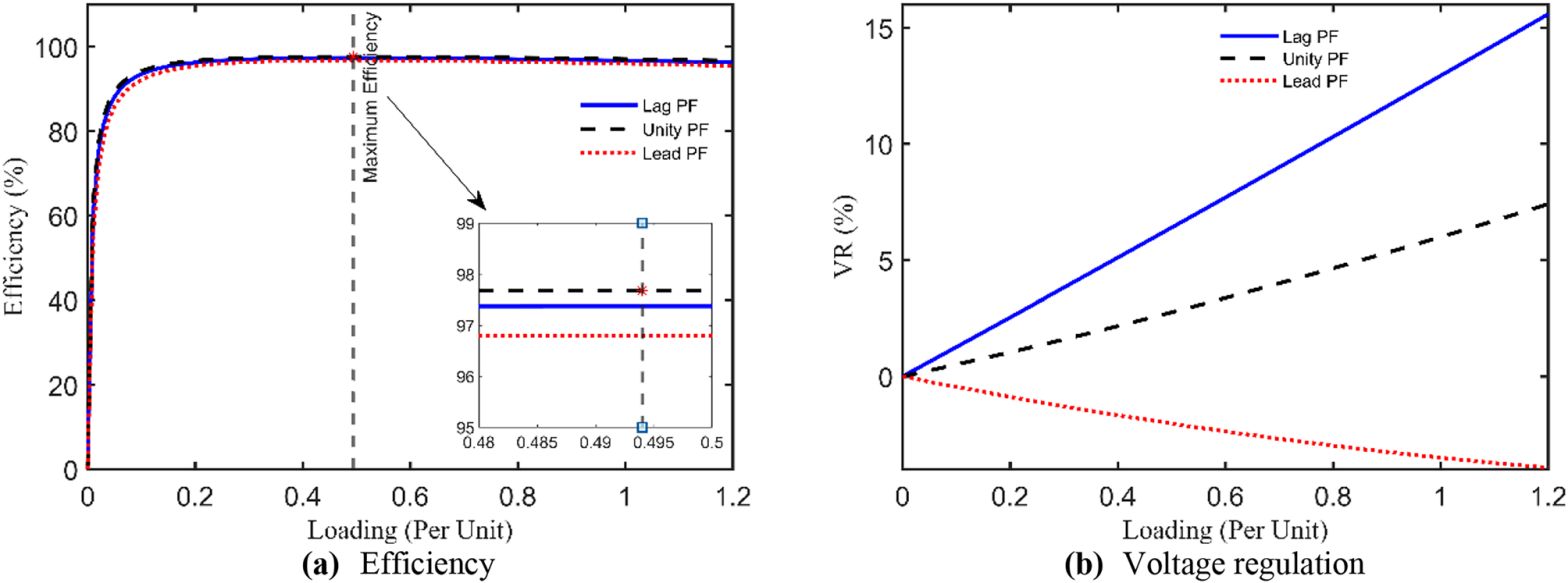
4 kVA TX steady state evaluation using IAEO.

4 kVA single-phase TX is rated at 250/125 V and 50 Hz with the following measured voltages and currents at full load: ${I}_{prms}=15.2825 A$, ${I}_{srms}=15.0782 A$, ${V}_{srms}=235.5967 V$, and ${\eta }_{\text{ms}}=94.07\%$. The 6 unknown parameters will be estimated using the IAEO along with the 3 rivals and compared with literature algorithms as reported in Table 4. Obviously, there are no violations in the reported values, however, IAEO and AEO attain the lowest possible value of the SAE compared to FA, PO, and EDO as indicated in Table 5. Moreover, Fig. 3 depicts the OF convergence using the nominated contenders showing the superior performance of the proposed IAEO. Additionally, IAEO achieves the lowest values for OF supplements as declared in Table 5 resulting in SAEs of 1.12e-5. In this regard, FA is considered as the best rival to IAEO with SAEs of 1.75e-3 while EDO loses the competition with SAEs of 0.0107. Afterwards, efficiency and VR curves are extracted for this TX using IAEO as declared in Fig. 4 using various loading power factors (PFs). Explicitly, VR accepts negative values for leading PFs as shown in Fig. 4(b) while the maximum efficiency occurs at 49.4% loading.

**Table 4:** Optimal parameters assessment of 4 kVA TX.

Optimizer	${{\varvec{R}}}_{{\varvec{p}}{\varvec{r}}}$[Ω]	${{\varvec{X}}}_{{\varvec{p}}{\varvec{r}}}$[Ω]	${{\varvec{R}}}_{{\varvec{s}}{\varvec{r}}}$[Ω]	${{\varvec{X}}}_{{\varvec{s}}{\varvec{r}}}$[Ω]	${{\varvec{R}}}_{{\varvec{c}}{\varvec{r}}}$[Ω]	${{\varvec{X}}}_{{\varvec{m}}{\varvec{r}}}$[Ω]
**IAEO**	**0.2924**	**0.8683**	**0.4914**	**1.3726**	**1353.9173**	**909.6498**
AEO	0.5253	0.8429	0.2909	0.9205	1646.2365	331.9101
FA	0.4045	0.9889	0.3950	0.9641	1477.0172	440.9090
PO	0.7325	0.1020	0.1005	1.8242	1888.2106	432.3580
EDO	0.1000	1.9967	0.6728	0.1699	1263.3860	899.7406
PSO^36^	0.4870	0.2990	0.3260	1.7560	1530.0000	621.0000
FBI^36^	0.4140	0.1722	0.4233	1.7250	1508.0000	653.0000
ICA^39^	0.4300	0.2020	0.3940	2.5000	1200.0000	700.0000
GSA^39^	0.4250	0.2030	0.4150	2.3990	1426.0000	750.3000
GA^38^	0.5980	0.2260	0.3360	1.9570	1410.0000	707.0000
JSO^41^	0.4050	0.2050	0.3950	1.9870	1520.0000	712.0000

**Table 5:** SAEs and OF complementary values of 4 kVA TX.

Variable	**IAEO**	AEO	FA	PO	EDO	PSO^36^	FBI^36^	JSO^41^	ICA^39^	GSA^39^	GA^38^
${I}_{prms}$	15.2825										
${I}_{pr}$	**15.2825**	15.2825	15.2842	15.2767	15.2778	15.2824	15.2820	15.2825	15.2449	15.2091	15.1714
$\left|{I}_{pr}-{I}_{prms}\right|$	**0.0000**	0.0000	1.668e-3	5.77e-3	4.666e-3	6.54e-4	6.322e-8	0.0000	0.0376	0.0734	0.1111
${I}_{srms}$	15.0782										
${I}_{sr}$	**15.0782**	15.0782	15.0782	15.0782	15.0781	15.0782	15.0782	15.0782	14.9881	15.2091	14.9574
$\left|{I}_{sr}-{I}_{srms}\right|$	**1.12e-5**	1.12e-5	1.3e-5	5e-6	1.26e-4	0.0000	4.577e-8	0.0000	0.0901	0.1309	0.1208
${V}_{srms}$	235.5967										
${V}_{sr}$	**235.5967**	235.5967	235.5967	235.5970	235.5949	235.5968	235.5967	235.5967	234.1890	234.2083	233.7090
$\left|{V}_{sr}-{V}_{srms}\right|$	**0.0000**	0.0000	2.8e-5	2.58e-4	1.796e-3	4.244e-5	7.152e-7	0.0000	1.4077	1.3884	1.8877
${\eta }_{ms}$	94.07%								NR		
$\eta$	**94.07%**	94.07%	94.07%	94.068%	94.0659%	94.01%	94.32%	94.08%			
$\eta -{\eta }_{ms}$	**0.0000**	0.0000	4e-5	1.025e-3	4.13e-3	0.0640	0.2550	0.0106			
**SAEs**	**1.12e-5**	1.12e-5	1.75e-3	7.058e-3	0.0107	0.0647	0.2550	0.0106			

NR = not reported.

A closer look at the convergence patterns demonstrated in Fig. 3 for AEO and IAEO, the reader may note that the IAEO accelerate faster to the final best value in only 50 cycles.

### Study case 2: 15 kVA

**Fig. 5:**
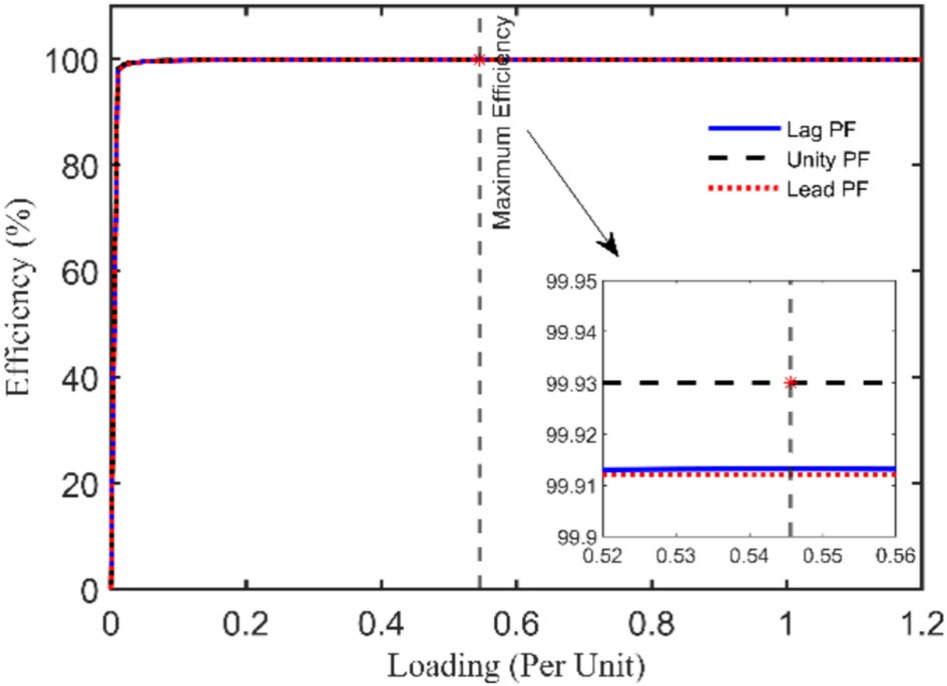
OF convergence trend of 15 kVA TX.

**Fig. 6:**
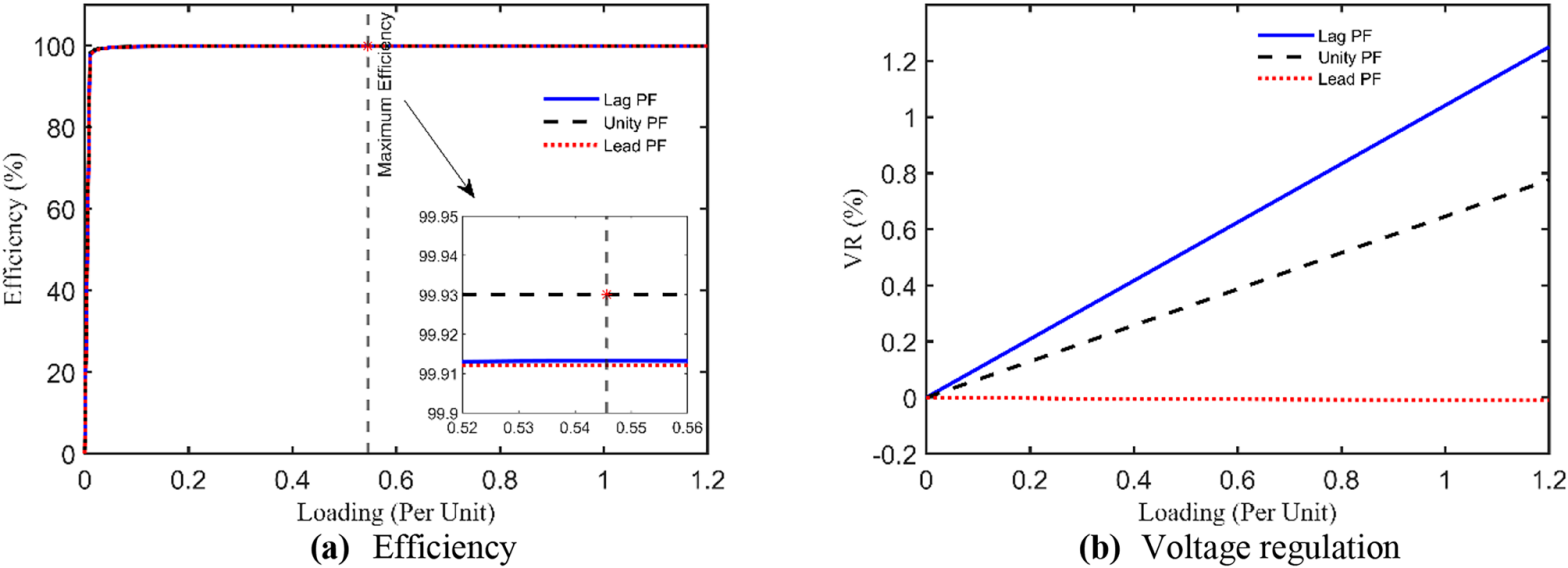
15 kVA TX steady state evaluation using IAEO.

The technical specs of this TX are 2400/240 V, 50 Hz, single-phase with the following measured currents and voltages at full load: ${I}_{prms}=6.2 A$, ${I}_{srms}=6.2 A$, and ${V}_{srms}=2383.8 V$. Table 6 announces the optimal unknown parameters of this test case using IAEO, FA, PO, and EDO with other reported results from the literature. Moreover, it has been revealed that IAEO attains the minimum OF value among all algorithms as indicated in Table 7 with SAEs of 0.0322. In this case, EDO is deemed as the best rival to IAEO by achieving SAEs of 0.0328 while FA loses the competition by 0.0424. In this context, Fig. 5 captures the OF convergence using the 5 executed algorithms showing a similar performance during the iterations process. It is worth mentioning that the proposed IAEO gains the best OF value compared to other referred work such as PSO, ICA, GSA, GA, COA, and AHO. Moreover, Fig. 6 expresses the steady state evaluation of this TX by printing the efficiency and VR CCs. The maximum efficiency arises at 54.5% loading factor meanwhile the VR CCs have almost flat behavior with leading PF loads.

**Table 6:** Optimal parameters assessment of 15 kVA TX.

Optimizer	${{\varvec{R}}}_{{\varvec{p}}{\varvec{r}}}$[Ω]	${{\varvec{X}}}_{{\varvec{p}}{\varvec{r}}}$[Ω]	${{\varvec{R}}}_{{\varvec{s}}{\varvec{r}}}$[Ω]	${{\varvec{X}}}_{{\varvec{s}}{\varvec{r}}}$[Ω]	${{\varvec{R}}}_{{\varvec{c}}{\varvec{r}}}$[Ω]	${{\varvec{X}}}_{{\varvec{m}}{\varvec{r}}}$[Ω]
**IAEO**	**2.3651**	**3.2776**	**0.1000**	**0.1000**	**200,000.0000**	**10,000.0000**
AEO	1.9523	2.4121	0.5431	1.3192	199,969.0161	9982.9408
FA	1.0655	2.1706	1.3963	1.6602	162,431.2497	6609.4549
PO	1.5982	2.6098	0.8770	2.3131	200,000.0000	10,000.0000
EDO	0.2632	4.5433	2.1389	0.3929	200,000.0000	10,000.0000
PSO^51^	2.2500	4.0820	2.2000	1.8526	99,517.0000	9009.0000
ICA^39^	2.0000	3.0000	1.8000	2.0000	120,000.0000	9200.0000
GSA^39^	2.0000	3.1100	1.8100	2.2600	104,281.0000	9094.8700
GA^51^	2.7600	3.4140	1.6800	1.8460	97,001.0000	8951.0000
COA^39^	1.9854	2.6117	1.4851	1.5203	131,010.0000	10,074.0000

**Table 7:** SAEs and OF complementary values of 15 kVA TX.

Variable	**IAEO**	AEO	FA	PO	EDO	PSO^51^	ICA^39^	GSA^39^	GA^51^	COA^39^	AHO^30^
${I}_{prms}$	6.2000										
${I}_{pr}$	**6.2244**	6.2252	6.2346	6.2258	6.2247	6.1979	6.2051	6.2081	6.1993	6.2079	6.2257
$\left|{I}_{pr}-{I}_{prms}\right|$	**0.0244**	0.0252	0.0346	0.0258	0.0247	0.0021	0.0051	0.0081	0.0007	0.0079	0.0257
${I}_{srms}$	6.2000										
${I}_{sr}$	**6.2078**	6.2078	6.2078	6.2078	6.2078	6.1672	6.1784	6.1781	6.1678	6.1843	6.2078
$\left|{I}_{sr}-{I}_{srms}\right|$	**0.0078**	0.0078	0.0078	0.0078	0.0078	0.0329	0.0216	0.0219	0.0322	0.0157	0.0078
${V}_{srms}$	2383.8000										
${V}_{sr}$	**2383.8000**	2383.8000	2383.8000	2383.7999	2383.8004	2371.1000	2375.5000	2375.3000	2371.4000	2377.7000	2383.8000
$\left|{V}_{sr}-{V}_{srms}\right|$	**0.0000**	0.0000	1e-6	4.3e-5	3.67e-4	12.7000	8.3000	8.5000	12.4000	6.1000	0.0000
**SAEs**	**0.0322**	0.0330	0.0424	0.0336	0.0328	12.7350	8.3267	8.5300	12.4205	6.1236	0.0335

### Study case 3: 112.5 kVA

**Fig. 7:**
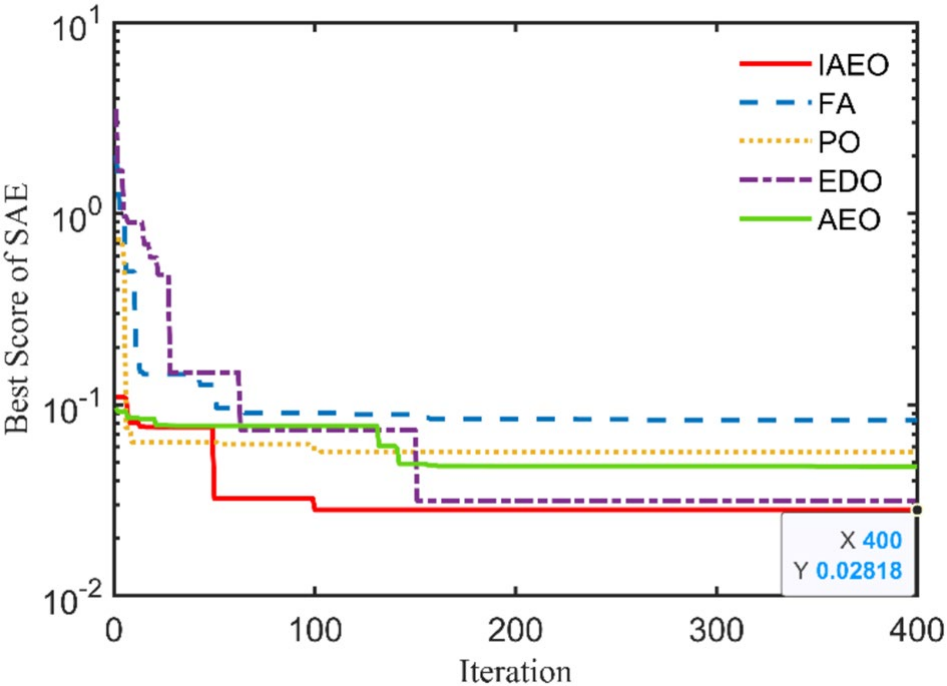
OF convergence trend of 112.5 kVA TX.

**Fig. 8:**
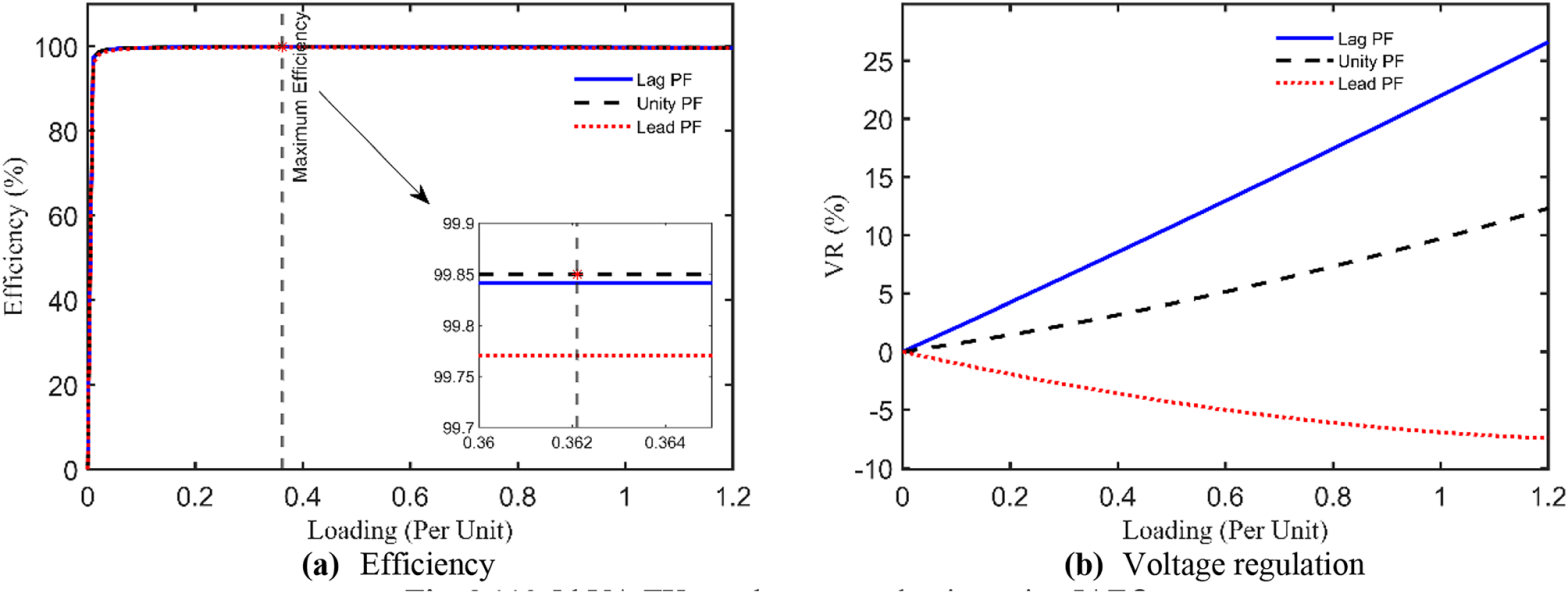
112.5 kVA TX steady state evaluation using IAEO.

This TX has the following nameplate data: 112.5 kVA, 13,200/440 V, 60 Hz, single-phase with these measured currents and voltage: ${I}_{prms}=7.9032 A$, ${I}_{srms}=7.7484 A$, and ${V}_{srms}=\text{12,000.7485} V$. The obtained optimal parameters are tabulated in Table 8 with ${R}_{pr}=32.2951 \Omega$, ${X}_{pr}=101.3391 \Omega$, ${R}_{sr}=70 \Omega$, ${X}_{sr}=300 \Omega$, ${R}_{cr}=200 k\Omega$, and ${X}_{mr}=40 k\Omega$ using the IAEO. Additionally, Table 9 manifests the preeminence of IAEO over other competitors by accomplishing SAEs of 0.0282 with a speedy convergence CCs as shown in Fig. 7. It is worthy to say that EDO is the best rival to IAEO by attaining SAEs of 0.0315 while FA and PO attain SAEs of 0.0833 and 0.0564 respectively. Even though, IAEO wins the overall competition by fulfilling a percentage reduction of the OF value by 11.7% compared to EDO. Additionally, the OF complementary values are the least among other optimizers as indicated in Table 9. Specifically, Fig. 8 shows the steady state fingerprint of this TX using the proposed IAEO when loading with diverse loading types. Its peak efficiency takes place at about 36.2% loading regardless of the PF type. Moreover, the VR CCs seems to be more intensive in the case of lag PF that may reach 20% for full loading condition.

**Table 8:** Optimal parameters assessment of 112.5 kVA TX.

Optimizer	${{\varvec{R}}}_{{\varvec{p}}{\varvec{r}}}$[Ω]	${{\varvec{X}}}_{{\varvec{p}}{\varvec{r}}}$[Ω]	${{\varvec{R}}}_{{\varvec{s}}{\varvec{r}}}$[Ω]	${{\varvec{X}}}_{{\varvec{s}}{\varvec{r}}}$[Ω]	${{\varvec{R}}}_{{\varvec{c}}{\varvec{r}}}$[Ω]	${{\varvec{X}}}_{{\varvec{m}}{\varvec{r}}}$[Ω]
**IAEO**	**32.2951**	**101.3319**	**70.0000**	**300.0000**	**200,000.0000**	**40,000.0000**
AEO	40.4405	163.3900	67.1608	204.5046	200,000.2812	40,724.6244
FA	49.9795	222.3930	44.4576	204.5535	275,701.5476	64,646.8383
PO	60.5766	100.5668	50.1858	264.9142	291,774.2327	40,656.0427
EDO	30.0000	109.3497	70.0000	300.0000	200,000.0000	40,000.0000

**Table 9:** SAEs and OF complementary values of 112.5 kVA TX.

Variable	**IAEO**	AEO	FA	PO	EDO
${I}_{prms}$	7.9032				
${I}_{pr}$	**7.8750**	7.8556	7.8199	7.8470	7.8750
$\left|{I}_{pr}-{I}_{prms}\right|$	**0.0282**	0.0476	0.0833	0.0562	0.0282
${I}_{srms}$	7.7484				
${I}_{sr}$	**7.7484**	7.7484	7.7484	7.7484	7.7484
$\left|{I}_{sr}-{I}_{srms}\right|$	**1.7e-5**	1.7e-5	1.7e-5	1.7e-5	1.5e-5
${V}_{srms}$	12,000.7485				
${V}_{sr}$	**12,000.7485**	12,000.7485	12,000.7485	12,000.7487	12,000.7452
$\left|{V}_{sr}-{V}_{srms}\right|$	**0.0000**	0.0000	7e-6	2.04e-4	0.0033
**SAEs**	**0.0282**	0.0476	0.0833	0.0564	0.0315

### Study case 4: 167 kVA

**Fig. 9:**
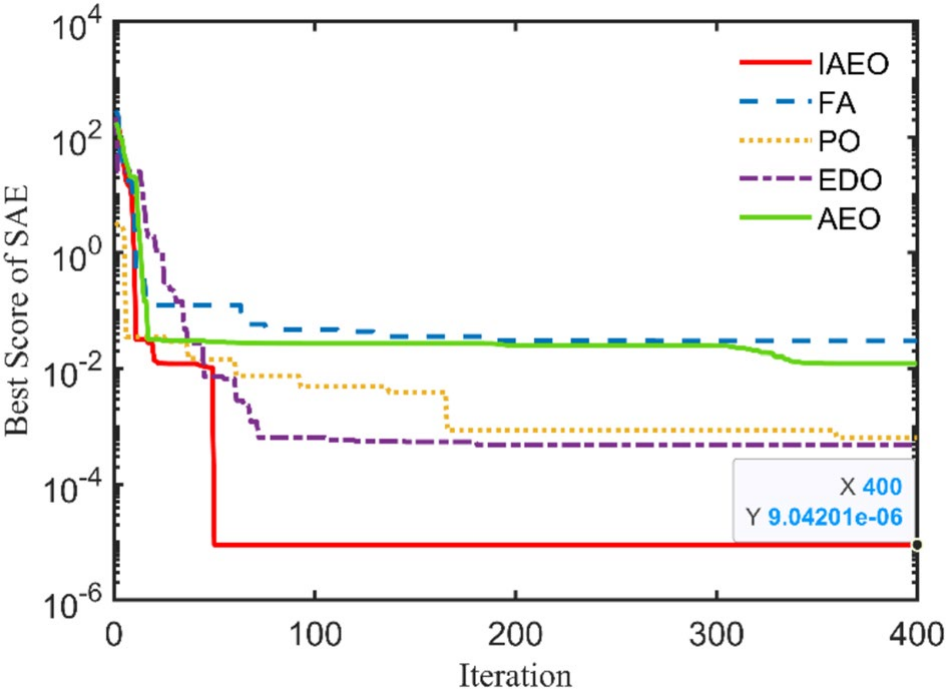
OF convergence trend of 167 kVA TX.

**Fig. 10:**
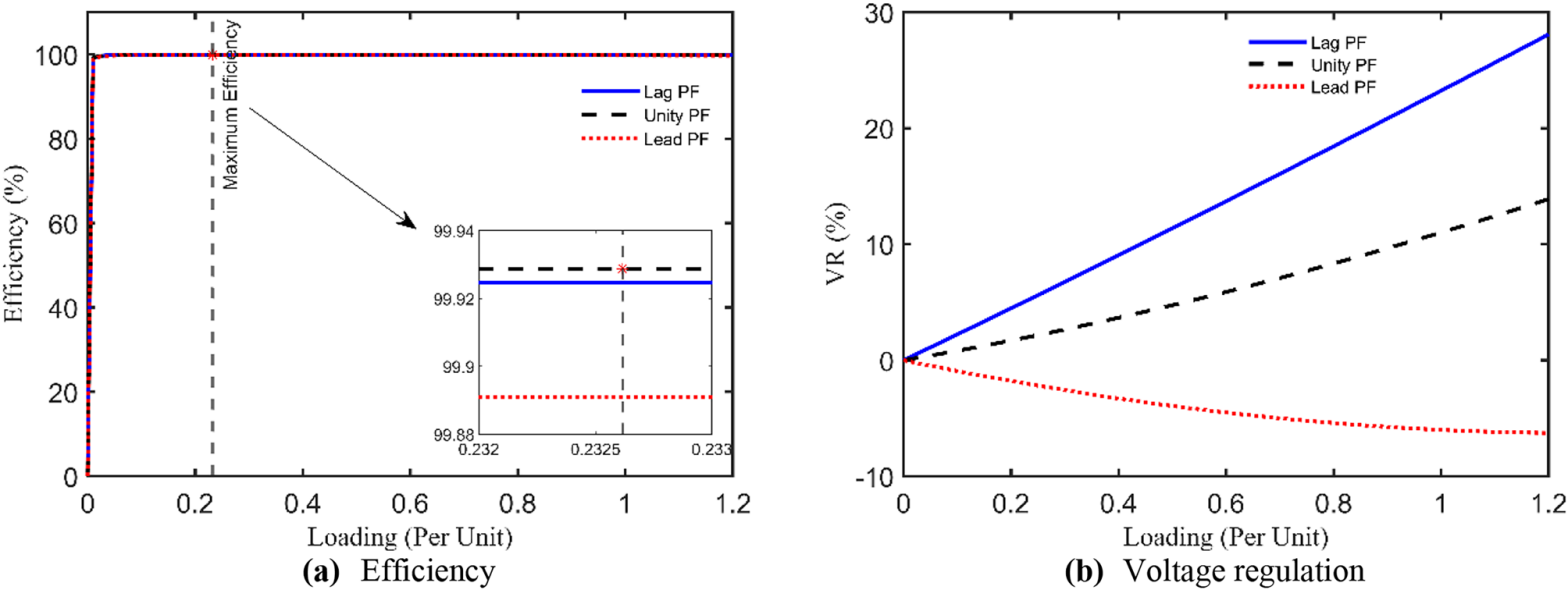
167 kVA TX steady state evaluation using IAEO.

This single-phase TX has these typical nameplate ratings: 167 kVA, 11,400/240 V, 60 Hz with the measured currents and voltages of ${I}_{prms}=13.2533 A$, ${I}_{srms}=13.1642 A$, and ${V}_{srms}=\text{10,244.4207} V$. The optimal unknown parameters of this TX are reported in Table 10 using the four executed algorithms. The reached results using IAEO are ${R}_{pr}=30.2025 \Omega$, ${X}_{pr}=100.4757 \Omega$, ${R}_{sr}=30.6067 \Omega$, ${X}_{sr}=105.4181 \Omega$, ${R}_{cr}=200.835 k\Omega$, and ${X}_{mr}=41.174 k\Omega$. Table 11 declares the dominance of IAEO over other challengers by attaining SAEs of 9e-6 with the excellence convergence CCs as shown in Fig. 9. Clearly, IAEO outperforms all algorithms by achieving a great percentage reduction of the OF value even when it is compared to the best rival (i.e. EDO). In this study case, FA loses the competition when accomplishing the highest OF value of 0.0296. In this regard, Fig. 10 captures the steady state performance of this large single-phase TX showing the trend of efficiency and VR CCs for various loads. Notably, the peak efficiency occurs at a lower value of loading almost 23.3% irrespective of the PF type. Moreover, the VR negative trend may reach −8% when this TX is loaded by its full loading leading PF.

**Table 10:** Optimal parameters assessment of 167 kVA TX.

Optimizer	${{\varvec{R}}}_{{\varvec{p}}{\varvec{r}}}$[Ω]	${{\varvec{X}}}_{{\varvec{p}}{\varvec{r}}}$[Ω]	${{\varvec{R}}}_{{\varvec{s}}{\varvec{r}}}$[Ω]	${{\varvec{X}}}_{{\varvec{s}}{\varvec{r}}}$[Ω]	${{\varvec{R}}}_{{\varvec{c}}{\varvec{r}}}$[Ω]	${{\varvec{X}}}_{{\varvec{m}}{\varvec{r}}}$[Ω]
**IAEO**	**30.2025**	**100.4757**	**30.6067**	**105.4181**	**200,835.2005**	**41,174.6208**
AEO	31.3754	102.1184	30.4967	101.8033	200,000.1627	59,570.0304
FA	30.9872	102.0069	30.8499	103.5172	258,406.5615	77,124.8567
PO	30.0000	108.1354	30.0000	100.1689	200,000.0000	40,000.0000
EDO	32.1847	100.0000	30.0000	100.0000	200,000.0000	40.000.0000

**Table 11:** SAEs and OF complementary values of 167 kVA TX.

Variable	**IAEO**	AEO	FA	PO	EDO
${I}_{prms}$	13.2533				
${I}_{pr}$	**13.2533**	13.2410	13.2237	13.2529	13.2528
$\left|{I}_{pr}-{I}_{prms}\right|$	**0.0000**	0.0123	0.0296	0.0004	0.0005
${I}_{srms}$	13.1642				
${I}_{sr}$	**13.1642**	13.1642	13.1642	13.1642	13.1642
$\left|{I}_{sr}-{I}_{srms}\right|$	**9e-6**	9e-6	9e-6	9e-6	9e-6
${V}_{srms}$	10,244.4207				
${V}_{sr}$	**10,244.4207**	10,244.4207	10,244.4207	10,244.4205	10,244.4207
$\left|{V}_{sr}-{V}_{srms}\right|$	**0.0000**	0.0000	5e-6	2.19e-4	5e-6
**SAEs**	**9e-6**	0.0123	0.0296	6.36e-4	4.77e-4

It is well known that $X/R$ defines the ratio between the equivalent reactance to the equivalent resistance, while ${I}_{o}$ denotes the percentage of the no-load current with respect to the nominal current. Hereinafter, the steady state CCs calculations are highlighted in Table 12 for the 4 test cases using the implemented algorithms. The results obtained are almost identical for each study case which proves the efficacy of the optimization methodology. It is concluded that 15 kVA TX reaches its peak efficiency at a higher percentage loading while the 167 kVA TX reaches the peak efficiency at the lower percentage loading. Obviously, the largest TX (i.e. 167 kVA) has the minimum no load current percentage with an average value of 2% using the 4 optimizers.

**Table 12:** Performance assessments under normal operating conditions.

**Case**	**Optimizer**	${{\varvec{P}}}_{{\varvec{c}}{\varvec{u}}{\varvec{f}}{\varvec{l}}}({\varvec{W}})$	${{\varvec{P}}}_{\mathbf{c}\mathbf{o}\mathbf{r}\mathbf{e}}({\varvec{W}})$	${\varvec{\varepsilon}}(\boldsymbol{\%})$	${{\varvec{Z}}}_{{\varvec{T}}{\varvec{X}}{\varvec{r}}}(\boldsymbol{\%})$	${\varvec{X}}/{\varvec{R}}$	${{\varvec{I}}}_{{\varvec{o}}}(\boldsymbol{\%})$
**Case 1: 4 kVA**	IAEO	180.0032	43.9318	49.4026	15.1936	2.8592	2.1135
	AEO	188.8339	35.1011	43.1142	12.4364	2.1604	4.8344
	FA	184.2867	39.6466	46.3827	13.5055	2.4428	3.7475
	PO	193.8004	30.1762	39.4598	13.4315	2.3124	3.7075
	EDO	176.2939	47.8034	52.0728	14.7217	2.8037	2.1948
**Case 2: 15 kVA**	IAEO	95.4855	28.4273	54.5631	1.0889	1.3701	3.8356
	AEO	96.5851	28.4977	54.3187	1.1690	1.4953	3.8461
	FA	95.2274	35.2396	60.8323	1.1859	1.5561	5.8108
	PO	95.7406	28.5435	54.6016	1.4349	1.9889	3.8425
	EDO	92.6252	28.7299	55.6933	1.4296	2.0550	3.8558
**Case 3: 112.5 kVA**	IAEO	6205.4784	813.6686	36.2106	26.7409	3.9233	4.1299
	AEO	6527.8282	796.4480	34.9297	24.7487	3.4191	4.0260
	FA	5725.4404	561.9000	31.3275	28.2326	4.5210	2.5288
	PO	6743.0986	540.5407	28.3129	24.6575	3.2997	3.9745
	EDO	6063.1407	813.6682	36.6332	27.2073	4.0935	4.1299
**Case 4: 167 kVA**	IAEO	10,609.0680	574.0603	23.2616	27.5874	3.3859	2.0086
	AEO	10,785.8468	575.6540	23.1022	27.3838	3.2959	1.4194
	FA	10,764.8229	446.1609	20.3583	27.5796	3.3236	1.0987
	PO	10,468.0520	574.6725	23.4303	27.8556	3.4717	2.0624
	EDO	10,851.7306	574.6433	23.0118	26.9138	3.2162	2.0623

### Further validations and statistical measures

**Fig. 11:**
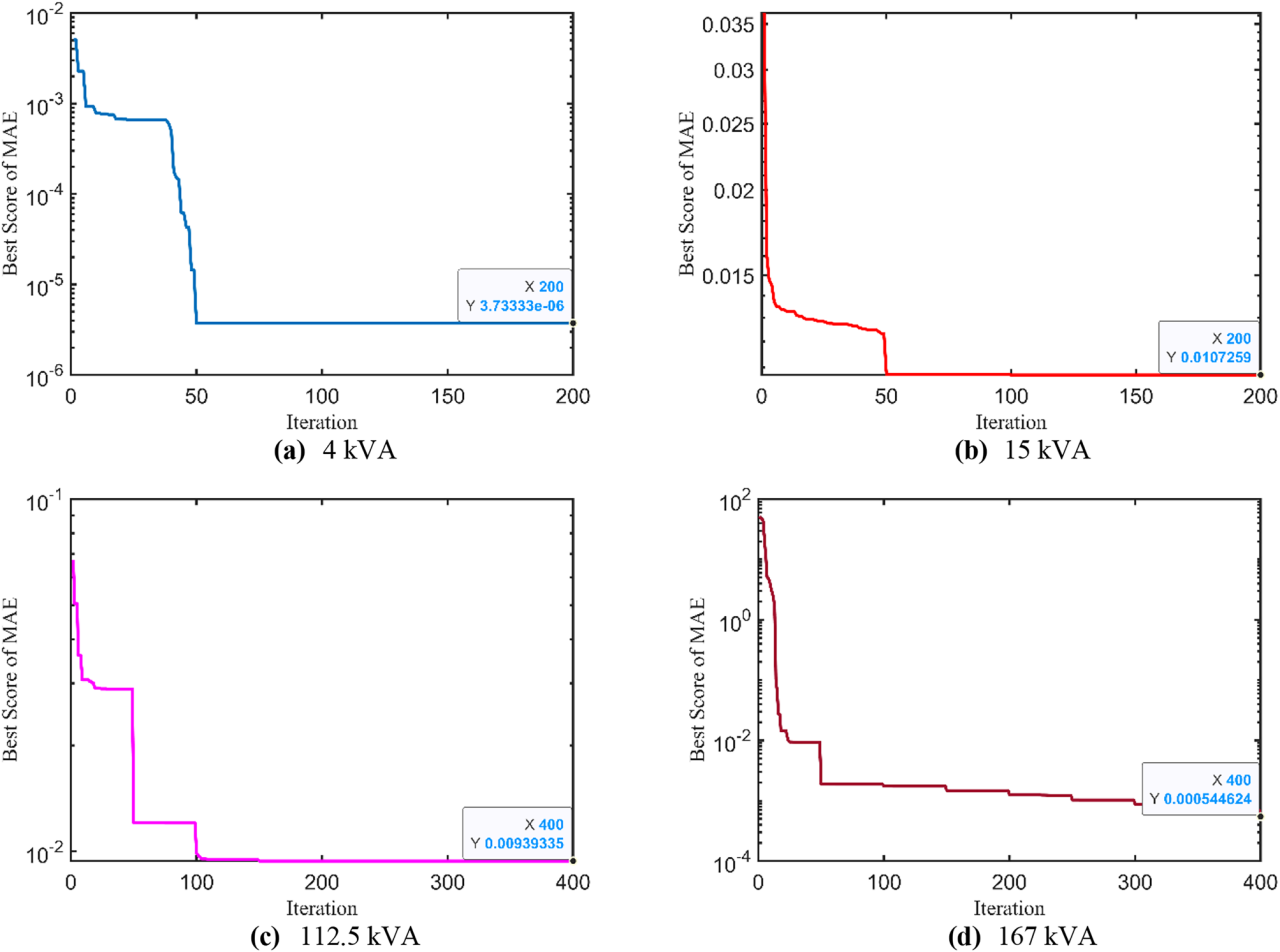
MAE OF convergence trend using IAEO.

**Fig. 12:**
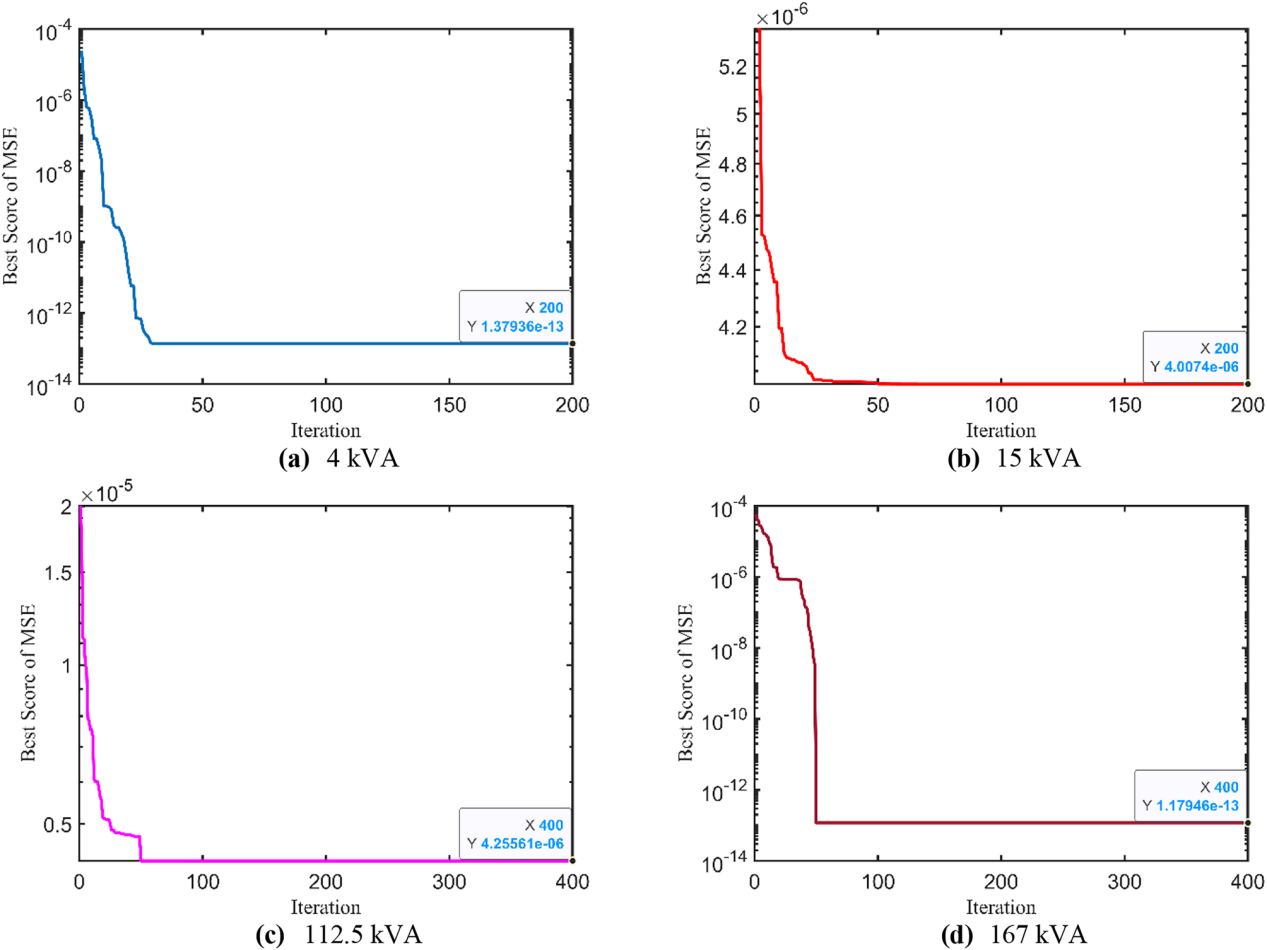
MSE OF convergence trend using IAEO.

Afterwards, the proposed IAEO-based methodology is validated using various OFs such as MAE and MSE. As reported in Table 13, the optimal parameters of all test cases with the corresponding OF are tabulated. Furthermore, Fig. 11 depicts the OF convergence of MAE metric while Fig. 12 shows the OF trend of MSE metric using the IAEO. Analyzing some results, optimal unknown parameters are very close using both metrices as compared to the original OF (i.e. SAE) especially, for the largest TXs. However, there are little variations in the obtained parameters of the 1^st^ test case when modelling different OF. Finally, whenever the OF changes, the optimal parameters can be efficiently recalculated and expected outcomes achieved.

**Table 13:** Optimal parameters assessment considering other OF formulas using IAEO.

**Metric**	Case	${{\varvec{R}}}_{{\varvec{p}}{\varvec{r}}}$**[Ω]**	${{\varvec{X}}}_{{\varvec{p}}{\varvec{r}}}$**[Ω]**	${{\varvec{R}}}_{{\varvec{s}}{\varvec{r}}}$**[Ω]**	${{\varvec{X}}}_{{\varvec{s}}{\varvec{r}}}$**[Ω]**	${{\varvec{R}}}_{{\varvec{c}}{\varvec{r}}}$**[Ω]**	${{\varvec{X}}}_{{\varvec{m}}{\varvec{r}}}$**[Ω]**	**OF**
**MAE**	Case 1: 4 kVA	0.2581	2.0980	0.3918	0.1023	1545.3457	215.9762	4e-6
	Case 2: 15 kVA	2.4550	1.2445	0.1000	0.1000	200,000.0000	10,000.0000	0.0107
	Case 3: 112.5 kVA	32.6042	100.2407	70.0000	300.0000	200,000.0000	40,000.0000	0.0094
	Case 4: 167 kVA	30.0001	100.0018	30.0094	109.7884	200,024.0263	44,753.1697	0.0005
**MSE**	Case 1: 4 kVA	0.1257	1.7619	0.3452	2.0744	1424.6098	934.8371	1.38e-13
	Case 2: 15 kVA	3.0081	3.7585	0.1000	0.1000	200,000.0000	10,000.0000	4.01e-6
	Case 3: 112.5 kVA	30.4974	100.3430	70.0000	300.0000	200,000.0000	40,000.0000	4.26e-6
	Case 4: 167 kVA	30.4979	100.5144	30.9844	102.3991	201,025.5846	40,132.1264	1.18e-13

**Fig. 13:**
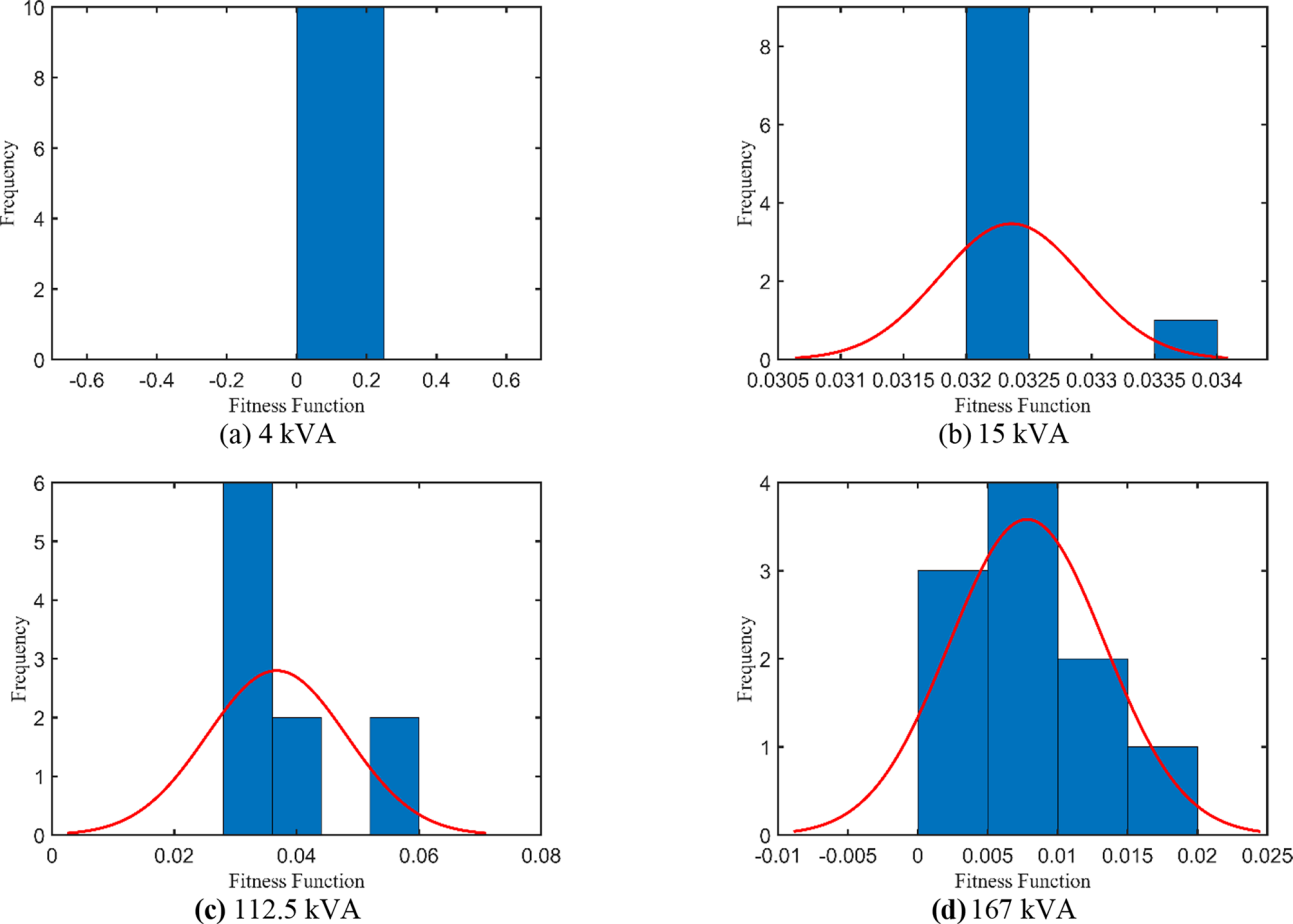
IAEO t-test analysis.

A closer look at Table 14, if the output value from statistics is larger than the value from t-distribution, the null hypothesis is rejected. Accordingly, Table 14 and Fig. 13 illustrate the above-mentioned statistical indices showing good accuracy in the simulation results.

**Table 14:** Statistical measures of the implemented optimizers.

**Case**	**Optimizer**	Min	Max	Mean	Median	SD	VR	CPU time (s)	h	p-value	ts	df
**Case 1: 4 kVA**	IAEO	1.12e-5	1.12e-5	1.12e-5	1.12e-5	0.0000	0.0000	1.2923	1	0.0000	Inf	9
	AEO	1.12e-5	1.5e-5	1.3e-5	1.34e-5	1.88e-6	3.5e-12	6.5719		0.0067	12.1347	
	FA	1.75e-3	0.1035	0.0405	0.0294	0.0353	0.0012	4.5992		0.0055	3.6342	
	PO	7.058e-3	0.0741	0.0341	0.0207	0.0259	6.7e-4	5.4663		0.0024	4.1612	
	EDO	0.0107	0.0637	0.0389	0.0417	0.0180	3.2e-4	3.1737		7.5e-5	6.8446	
**Case 2: 15 kVA**	IAEO	0.0322	0.0340	0.0324	0.0322	5.8e-4	3.3e-7	1.4298		0.0000	177.9471	
	AEO	0.0330	0.0338	0.0334	0.0334	4.08e-4	1.6e-7	6.4402		5e-5	141.7545	
	FA	0.0424	0.0493	0.0446	0.0439	2.1e-3	4.4e-6	8.6909		0.0000	66.9398	
	PO	0.0336	0.0444	0.0376	0.0362	4.1e-3	1.7e-5	6.1595		0.0000	28.7300	
	EDO	0.0328	0.0368	0.0344	0.0341	1.2e-3	1.5e-6	4.5553		0.0000	89.3921	
**Case 3: 112.5 kVA**	IAEO	0.0282	0.0593	0.0367	0.0301	0.0114	1.3e-4	4.4464		3e-6	10.1855	
	AEO	0.0476	0.0706	0.0614	0.0660	0.0122	1.5e-4	12.1194		0.0129	8.7279	
	FA	0.0833	0.0952	0.0883	0.0879	0.0042	1.8e-5	9.4908		0.0000	66.7527	
	PO	0.0564	0.0857	0.0747	0.0779	0.0093	8.7e-5	9.8527		0.0000	25.3095	
	EDO	0.0315	0.0776	0.0578	0.0614	0.0158	2.5e-4	6.9659		1e-6	11.5729	
**Case 4: 167 kVA**	IAEO	9e-6	0.0176	0.0078	6.6e-3	5.6e-3	3.1e-5	5.5264		0.0016	4.4392	
	AEO	0.0123	0.0181	0.0155	0.0161	2.9e-3	8.7e-6	12.7737		0.0119	9.0884	
	FA	0.0296	0.0393	0.0346	0.0344	3.3e-3	1.1e-5	9.1849		0.0000	32.8652	
	PO	6.36e-4	0.0141	0.0041	2.56e-3	4.4e-3	1.9e-5	11.1582		0.0162	2.9514	
	EDO	4.77e-4	0.0024	0.0015	1.34e-3	6.6e-4	4.3e-7	7.3837		5.4e-5	7.1408

Despite the promising results, this study has certain limitations. The optimization process relies on algorithm-specific parameters, which require careful tuning for effective performance. Additionally, the stochastic nature of metaheuristic methods may result in variability across runs. The computational time and scalability of the proposed approach can also pose challenges for larger or real-time systems. Moreover, the transformer models used are based on standard assumptions and simulations, with no direct real-world validation conducted. Future work may address these aspects by incorporating adaptive tuning, hybrid optimization methods, and practical field testing.

## Conclusions

This paper presents a forward step in accurate estimation of the 6 unknown parameters of distribution transformers with several ratings. This is achieved by utilizing a novel enhanced version of the AEO enhancing the efficiency of the consumption phase by incorporating sinusoidal-based one’s technique. This way, IAEO succeeds in obtaining the lowest possible value of SAEs among all other literature methods such as the well-established GA and PSO. Additionally, FA, PO, and EDO are tested along with the proposed IAEO over the 4 study cases showing the superior performance of IAEO. For instance, 1.12e-5 is the best OF value attained by IAEO compared to 1.75e-3 achieved by FA in the 4 kVA TX, while in the 112.5 kVA TX, IAEO gains 11.7% reduction in the SAEs compared to the EDO. Furthermore, efficiency and voltage regulation CCs are extracted for all study cases at different loading conditions. It has been revealed that the largest TX with a size of 167 kVA exhibits a notable increment in the terminal voltage by 8% when it is fully loaded with its rated leading PF. Conversely, the VR experiences a flat behavior as in the case of 15 kVA TX when it is loaded with rated PF. Considering other aspects, the peak efficiency takes place at 49.4% and 36.2% for 4 kVA and 112.5 kVA TXs respectively. Nevertheless, this study is confined to analyze TXs operation under steady-state conditions, without accounting for transient events. In future work, the proposed methodology will be validated and tested on large power TXs, taking into consideration the influence of let-through fault protection CCs.

## Supplementary Information

Supplementary Information.

## Data Availability

The datasets generated and/or analyzed during the current study are not publicly available due to intellectual property rights but are available from the corresponding author on reasonable request.

## Abbreviations

ABC: Artificial bee colony
AEO: Artificial ecosystem optimizer
AHO: Artificial hummingbird optimizer
AMQE: Average mean quadratic error
BOA: Blackhole optimization algorithm
CCs: Characteristic curves
COA: Coyote optimization algorithm
COT: Chaotic optimization technique
CSO: Crow search optimizer
DC: Direct current
DOA: Dandelion optimization algorithm
EDO: Exponential distribution optimizer
FA: Firefly algorithm
FBI: Forensic-based investigation optimizer
FIO: Forensic-based investigation optimizer
GA: Genetic algorithm
GNDO: Generalized normal distribution optimizer
GOA: Grasshopper optimization algorithm
GSA: Gravitational search algorithm
HOA: Hurricane optimization algorithm
IAEO: Improved artificial ecosystem optimizer
ICA: Imperialistic competitive algorithm
ICO: Imperialistic competitive optimizer
ITDO: Improved tasmanian devil optimizer
JSO: Jellyfish search optimizer
LB: Lower boundary
MOA: Metaheuristic optimization algorithm
MQE: Mean quadratic error
NR: Not reported
OF: Objective function
PE: Percentage error
PO: Political optimizer
PSO: Particle swarm optimization
PV: Photovoltaic
SAE: Sum of absolute error
SD: Standard deviation
SMO: Slime mould optimizer
SQAPE: Sum of quadratic absolute percentage error
SQE: Sum of quadratic error
SQRE: Sum of quadratic relative error
TX: Transformer
UB: Upper boundary
VR: Voltage regulation

## List of symbols

$C$: Consumption factor based on Levy flight
$$D$$: Decomposition strength
$e$: Decomposition direction factor
$h$: Strength factor in decomposition
${I}_{pr}$: Computed primary current (A)
${I}_{prms}$: Measured primary current (A)
${I}_{sr}$: Computed secondary current (A)
${I}_{srms}$: Measured secondary current (A)
$it$: Current iteration number
$I{T}_{m}$: Maximum number of iterations
$${N}_{p}$$: Population count
$$OF$$: Objective Function
${R}_{cr}$: Core loss resistance (Ω)
$${R}_{pr}$$: Primary resistance (Ω)
$${R}_{st}$$: Secondary resistance (Ω)
$SAE$: Sum of Absolute Error
$s,{s}_{1},{s}_{2},{s}_{3}, {s}_{4},{s}_{5},{s}_{6}$: Random numbers/vectors in range [0, 1]
${U}_{l}$*, *${L}_{l}$: Upper and lower bounds of decision variables
${V}_{sr}$: Computed secondary voltage (V)
${V}_{srms}$: Measured secondary voltage (V)
${X}_{1}\left(it\right)$: Global best position at iteration $it$
${X}_{i}\left(it\right)$: Individual position at iteration $it$
$${X}_{mr}$$: Magnetizing reactance (Ω)
$${X}_{pr}$$: Primary reactance (Ω)
${X}_{rd}$: Random candidate in the population
$${X}_{sr}$$: Secondary reactance (Ω)
$\eta$: Computed transformer efficiency (%)
${\eta }_{ms}$: Measured transformer efficiency (%)
${v}_{1}$, ${v}_{2}$: Random Gaussian-distributed variables
